# TAAR1 Expression in Human Macrophages and Brain Tissue: A Potential Novel Facet of MS Neuroinflammation

**DOI:** 10.3390/ijms222111576

**Published:** 2021-10-27

**Authors:** David A. Barnes, Dylan A. Galloway, Marius C. Hoener, Mark D. Berry, Craig S. Moore

**Affiliations:** 1 Department of Biochemistry, Faculty of Science, Memorial University of Newfoundland, 232 Elizabeth Ave, St. John’s, NL A1B 3X9, Canada; dab866@mun.ca (D.A.B.); mberry@mun.ca (M.D.B.); 2 Division of Biomedical Sciences, Faculty of Medicine, Memorial University of Newfoundland, 300 Prince Philip Drive, St. John’s, NL A1B 3V6, Canada; dgalloway@mun.ca; 3 Neuroscience, Ophthalmology and Rare Diseases DTA, pRED, Roche Innovation Center Basel, F. Hoffmann-La Roche, 4070 Basel, Switzerland; marius.hoener@roche.com

**Keywords:** multiple sclerosis, trace amines, trace amine-associated receptor 1, neuroinflammation

## Abstract

TAAR1 is a neuroregulator with emerging evidence suggesting a role in immunomodulation. Multiple sclerosis (MS) is an immune-mediated demyelinating disease of the central nervous system. Here, we investigate TAAR1 expression in human primary monocytes, peripherally-derived macrophages, and MS brain tissue. RT-qPCR was used to assess TAAR1 levels in MS monocytes. Using a previously validated anti-human TAAR1 antibody and fluorescence microscopy, TAAR1 protein was visualized in lipopolysaccharide-stimulated or basal human macrophages, as well as macrophage/microglia populations surrounding, bordering, and within a mixed active/inactive MS lesion. In vivo, TAAR1 mRNA expression was significantly lower in MS monocytes compared to age- and sex-matched healthy controls. In vitro, TAAR1 protein showed a predominant nuclear localization in quiescent/control macrophages with a shift to a diffuse intracellular distribution following lipopolysaccharide-induced activation. In brain tissue, TAAR1 protein was predominantly expressed in macrophages/microglia within the border region of mixed active/inactive MS lesions. Considering that TAAR1-mediated anti-inflammatory effects have been previously reported, decreased mRNA in MS patients suggests possible pathophysiologic relevance. A shift in TAAR1 localization following pro-inflammatory activation suggests its function is altered in pro-inflammatory states, while TAAR1-expressing macrophages/microglia bordering an MS lesion supports TAAR1 as a novel pharmacological target in cells directly implicated in MS neuroinflammation.

## 1. Introduction

Multiple sclerosis (MS) is an immune-mediated disease of the central nervous system (CNS) that results in demyelination and chronic neurodegeneration [1]. MS pathology is classified as a dysfunction in immunotolerance, wherein extravasation and activation of peripheral immune cells targeted against myelin antigens causes an inflammatory cascade, ultimately leading to demyelination, oligodendrocyte death, and axonal damage [2]. In addition to the infiltrating blood-derived immune cells, resident CNS cells, including microglia and astrocytes, are also activated and contribute to the pathogenesis and neurodegeneration [3]. Homeostatically, microglia function as the brain’s tissue-resident macrophages and are associated with injury repair and cytokine secretion [4], whereas, astrocytes function to regulate pH, direct fluid and ion flow, facilitate neurotransmitter transport, and help maintain the blood–brain barrier [5]. The identification of regulatory targets within CNS-resident cells and peripheral immune cells is key to the discovery of novel therapies and treatment strategies in MS.

Trace amines are bioactive amines endogenously present in vertebrate tissue and bodily fluids that selectively act on a class of G protein-coupled receptors (GPCRs) known as trace amine-associated receptors (TAARs); trace amines are classically regarded as consisting of p-tyramine, 2-phenylethylamine, tryptamine, and p-octopamine [6]. Of the different TAARs expressed in humans, the vast majority of research has focused on TAAR1 and its role in modulating neurotransmission within the CNS. With an established function of TAAR1 in the CNS and the availability of selective and safe TAAR1 agonists, pharmacologically targeting TAAR1 within the CNS has resulted in successful clinical trials for treatment of neurological disorders, such as schizophrenia [7,8], which also is accompanied by an inflammatory component [9]. In addition to the established role in the CNS, TAAR1 has also been associated with putative roles in regulation of the immune system including chemotactic migration, cytokine secretion, and differentiation of various immune cell populations [10]. However, these putative immunomodulatory effects have been less studied, particularly with respect to the CNS and its resident and infiltrating immune cells.

Within the peripheral immune system, TAAR1 mRNA is present in whole peripheral blood mononuclear cells and is upregulated in response to pro-inflammatory activation [11,12]. TAAR1 protein has also been identified in both malignant and normal human B cells [13]. In primary human B cells and immortalized B cell lines, TAAR1 protein expression is elevated following activation [13,14]. While support for the expression of TAAR1 within the peripheral immune system is consistent, there have been contradictory results reported with respect to the function of TAAR1. In mouse bone marrow-derived macrophages, TAAR1 mRNA has been shown to increase following treatment with the TAAR1 agonist p-tyramine, as well as under pro-inflammatory polarization induced by LPS, an effect mirrored by increased mRNA levels of pro-inflammatory cytokines including: IL-6, IL-1β, and TNF [15]. In contrast, TAAR1 has also been implicated in anti-inflammatory immune responses [16]. A TAAR1-mediated chemotactic migration of granulocytes towards trace amines has been reported, as well as induction of IgE secretion from B cells and IL-4 secretion from T cells [16]. These latter results suggest a TAAR1-mediated promotion of Th2 differentiation, consistent with TAAR1 agonism promoting anti-inflammatory responses [16].

Fewer studies have examined the role of TAAR1 in CNS-resident neuro-immune cells. TAAR1 agonists have been shown to influence key astrocytic responses including glutamate clearance thereby influencing the excitability of nearby neurons [17,18]. A retrospective meta-analysis suggested TAAR1 is expressed at very low levels in microglia [19], but no further research has been published in this area.

Given the potential for TAAR1 as a regulator of both peripheral and CNS-resident immune cell function, as well as the established interplay of both in MS, investigation into TAAR1 as a novel immunoregulator in MS represents an unexplored avenue in both trace amine and MS research.

The objectives of this study were to assess TAAR1 at both the mRNA and protein level in MS patient samples and controls. In the following communication, we demonstrate for the first time that TAAR1 levels are lower in MS patient monocytes, TAAR1 displays an altered sub-cellular localization in pro-inflammatory stimulated macrophages, and TAAR1 protein is prominently present within inflammatory regions of MS brain lesions. Together, these results suggest TAAR1 may represent a novel target and/or immunomodulator in MS pathogenesis. In addition to a novel contribution to MS research, our results provide the first demonstration of TAAR1 protein presence in human brain tissue.

## 2. Results

### 2.1. TAAR1 mRNA Expression Levels Are Variable in Whole Peripheral Blood Mononuclear Cells (PBMCs) Derived from MS Patients and Decreased in MS CD14+ Monocytes

**Figure 1:**
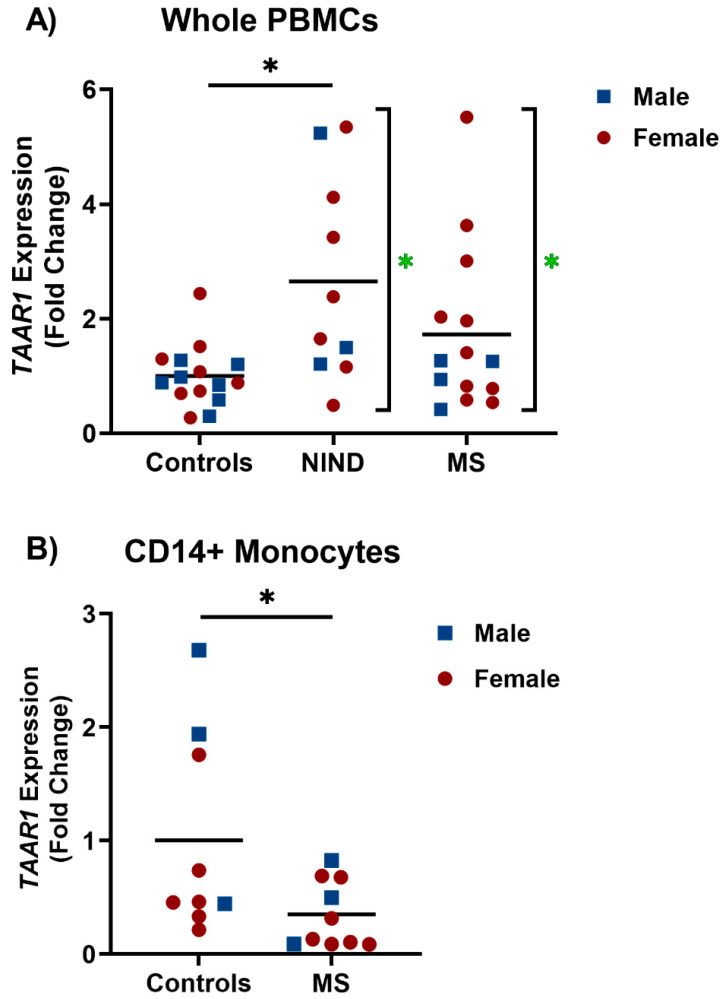
TAAR1 expression in multiple sclerosis (MS) whole peripheral blood mononuclear cells (PBMCs), CD14+ monocytes, and the respective controls. The ΔΔCT method was used to compare TAAR1 expression to the GAPDH housekeeping gene. (**A**) Brown-Forsythe test showed significant differences between-group variances (* *p* = 0.0284, F = 4.284). Significant effects were therefore determined by the Kruskal–Wallis test with between-group comparisons by Dunn’s post-hoc multiple comparison test (* *p* = 0.0168 for NIND vs. Controls, *p* = 0.4130 for MS vs. Controls). (**B**) Two-tailed unpaired *t* test showed a significant difference between the groups (* *p* = 0.0424, t = 2.194, df = 17). Blue squares denote individual male participants; red circles denote individual female participants.

RT-qPCR was used to measure TAAR1 expression levels in whole PBMCs from MS patients, non-inflammatory neurological disorder (NIND) patients, and healthy study participants (Figure 1A). Results were normalized to the mean of the healthy volunteer samples to determine fold change in relative TAAR1 expression. A significant increase in variance was seen in the MS and NIND patients when compared to the healthy controls (Brown-Forsythe test; * *p* = 0.0284, F = 4.284). While there was no significant change seen in the MS patients, TAAR1 levels were significantly higher in the NIND patients than the healthy controls (Kruskal–Wallis Test; *p* = 0.0284, F = 4.284 with Dunn’s post-hoc multiple comparison test; * *p* = 0.0168 for NIND vs. Controls, *p* = 0.4130 for MS vs. Controls). RT-qPCR was also used to measure TAAR1 levels in CD14+ monocytes from a different cohort of MS patients and healthy volunteers (Figure 1B) with a significant decrease in relative TAAR1 expression seen in MS patients (* *p* = 0.0424, t = 2.194, df = 17). In both whole PBMCs and CD14+ monocytes, cohorts were age- and sex-matched, with no significant effect of sex observed in either case.

### 2.2. TAAR1 Is Expressed in CD14-Derived Macrophages and Displays a Pronounced Shift out of the Nucleus Following LPS Stimulation

**Figure 2:**
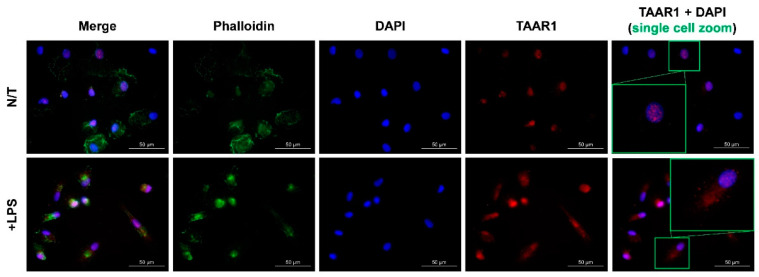
TAAR1 localization within basal and LPS-stimulated CD14+ monocyte-derived macrophages from healthy volunteers.

A validated and selective anti-TAAR1 antibody [20] was used to assess TAAR1 protein expression and localization within healthy CD14+ monocyte-derived macrophages under basal and LPS-stimulated conditions. Specificity of antibody binding was verified with a no-primary antibody control (Supplementary Figure S1). TAAR1 staining was observed almost exclusively within the nucleus under basal conditions (Figure 2). Following a 24-h LPS stimulation, phalloidin staining showed a cellular contraction consistent with the established amoeboid characteristics of macrophages [21]. In these LPS-stimulated cells, TAAR1 expression showed a pronounced expansion out of the nucleus to also include prominent cytoplasmic staining (Figure 2).

TAAR1 protein was visualized with a validated anti-human TAAR1 mouse primary antibody combined with a goat anti-mouse IgG AlexaFluor™ 594-conjugated secondary antibody (red). Nuclei were visualized via DAPI staining (blue) and actin with AlexaFluor™ 647-conjugated phalloidin (green). The last column contains the DAPI and anti-TAAR1 channels featuring a zoom on a single cell (green boxes). All images were taken with the Zeiss AX10 fluorescent microscope at 63× magnification generated in the built-in Zeiss software.

### 2.3. TAAR1 Protein Expression Is Present in the Inflamed Area of a Mixed Active/Inactive MS Lesion

**Figure 3:**
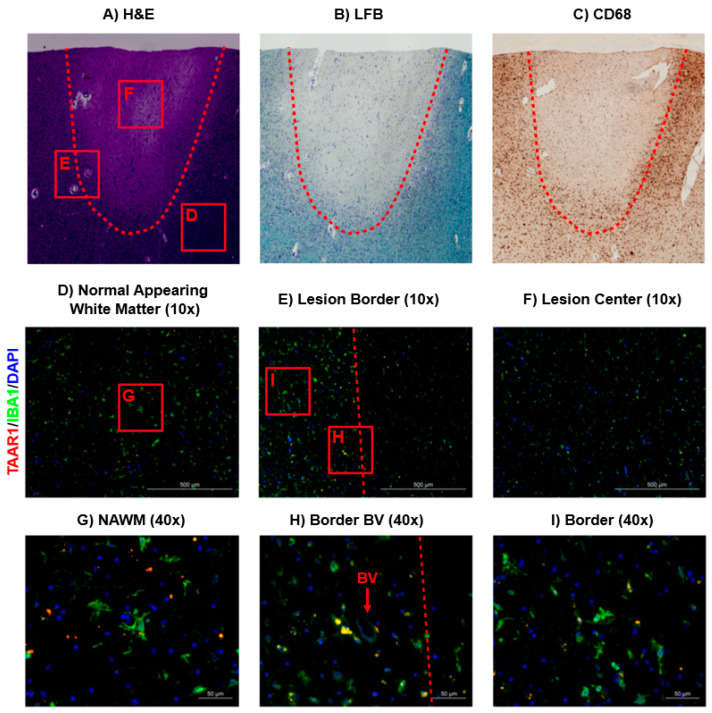
TAAR1 expression profile within and surrounding a mixed active/inactive MS lesion. Bright field images of a mixed active/inactive MS lesion were visualized using H&E (**A**), LFB (**B**), and CD68 (**C**) staining. Primary antibodies for TAAR1 and IBA-1 were used in combination with AlexaFluor™ 594(red) and AlexaFluor™ 647(green) conjugated secondary antibodies to visualize TAAR1 expression and microglia/macrophage localization, respectively (**D**–**I**). DAPI staining was used to identify nucleated cells (blue). Co-localization of IBA-1 and TAAR1 can be seen in yellow (**D**–**I**). Bright-field images were taken with the Cytation™ 5 Cell Imaging Multi-Mode Reader (BioTek). Fluorescent imaging was conducted with the Zeiss AX10 fluorescent microscope at 10× and 40× magnification and images generated in the built-in Zeiss software; Zen 2 Pro.

Within in situ specimens, CD68 was used as a marker for microglia/macrophages (Figure 3A) in combination with luxol fast blue (LFB) staining for myelin (Figure 3B) to identify a mixed active/inactive MS lesion via bright-field microscopy, as previously described in our lab [22]. Primary antibodies for TAAR1 and IBA-1 were then used to visualize TAAR1 expression and the corresponding microglia/macrophage populations (Figure 3D–I). Notably, TAAR1 expression was most visible in the normal appearing white matter (NAWM) and the border of the MS lesion (Figure 3D,E). Co-staining for IBA-1 and TAAR1 was primarily observed near the lesion border (yellow; Figure 3H,I), with TAAR1 staining particularly prominent in macrophages/microglia surrounding a blood vessel and possibly in the process of extravasation (BV; Figure 3H). Notably, TAAR1 sub-cellular distribution was diffuse in these macrophages/microglia, similar to the LPS-activated macrophages (Figure 2). While TAAR1 staining was visible in the NAWM near the lesion, there was a clear reduction in co-staining with IBA-1 (Figure 3G). When examining the lesion center, there was less TAAR1 staining and macrophages/microglia than the other regions examined (Figure 3F).

## 3. Discussion

Based on the established neuroregulatory [6] and emerging immunomodulatory properties of TAAR1 [10], we aimed to investigate whether TAAR1 could be a novel protein of interest in the pathophysiology of MS. Herein, we have provided the first systematic analysis of TAAR1 expression at both the mRNA and protein level in primary MS patient samples, from both the periphery and within the CNS. With an increasing interest in the myeloid cell compartment in the context of MS pathogenesis [4], our study focused on macrophages and their peripheral precursors, monocytes. RT-qPCR analyses of both whole MS patient PBMCs and CD14+ monocytes provided a potential clinical relevance for TAAR1 in MS and the basis for further subsequent systematic examination of the role of TAAR1 in human monocytes and macrophages.

Analyses of relative TAAR1 expression demonstrated no significant difference in TAAR1 mRNA levels in MS patient whole PBMCs compared to controls. However, in both the NIND and MS patient groups, there was a significant increase in variance compared to controls (Figure 1A). The multiple subtypes and fluidity associated with MS progression and relapse [23] could be related to this observed increase in variance, raising the possibility that increased TAAR1 expression is a biomarker of disease subtypes. Additionally, MS is prevalent in the global population at a female to male ratio of 2:1 [23], which was represented within our cohort. However, participant numbers left us unable to conclude any significant effect of sex in either whole PBMCs or CD14+ monocytes. With an increased sample size, the increased variation could result in significant differences observed, as seen in the NIND group (Figure 1A).

When assessing CD14+ monocytes, a significant decrease was observed when comparing TAAR1 mRNA levels in MS patients to healthy volunteers (Figure 1B). With conflicting reports of TAAR1 function in the immune system relating to the pro-inflammatory [15] or anti-inflammatory immune response [16], a decrease in TAAR1 mRNA levels in MS monocytes, a pro-inflammatory clinical indication, suggests TAAR1 may play a role in the maintenance and/or promoting of anti-inflammatory responses, at least in monocytes. That whole PBMCs trended toward an increase, which may be indicative of different functions of TAAR1 in different leukocyte populations. The MS patient monocytes and PBMCs used for this study were collected in treatment-naïve patients early in their disease course and as such there was no confounding factors relating to treatment status. In MS, the polarization of peripherally-derived and CNS-resident macrophages is directly involved in the periods of inflammatory demyelination and anti-inflammatory remyelination associated with relapse and remission periods [1,4]. Sample numbers did not allow analysis of whether expression levels were related to relapse or remission status at the time of blood sampling, and this is an area worth considering in future studies. Previous reports have associated TAAR1 activation with anti-inflammatory T cell differentiation [16]. If TAAR1 has a similar function in monocytes and monocyte-derived macrophages, lower TAAR1 mRNA could be indicative of decreased anti-inflammatory differentiation and/or increased demyelination.

In contrast to the common localization of GPCRs within the plasma membrane, reports of TAAR1 expression primarily describe an intracellular localization [20,24,25,26,27,28]. Using macrophages derived from human CD14+ peripheral monocytes, we observed TAAR1 localization under standard cell culture conditions and following pro-inflammatory stimulation. Under standard conditions, resting macrophages showed TAAR1 protein expression primarily localized to the nucleus, with pro-inflammatory stimulation showing a shift to a diffuse intracellular distribution (Figure 2), presumably cytoplasmic. Nuclear functions of GPCRs are an emerging area of therapeutic interest, with established functions in regulation of transcription, cell proliferation, cell migration, apoptosis, and angiogenesis [29]. With regards to TAAR1, nuclear sub-cellular distribution has previously been seen in some, but not all, breast cancer cell lines [26], although the functional relevance of this has yet to be determined. Altered sub-cellular localization of GPCRs has been previously reported to occur during immune cell activation [30]. For example, the sphingosine-1-phosphate receptor was reported to alter expression, localization, and function in stimulated and unstimulated T cells [30]. Following stimulation, the altered sub-cellular localization of TAAR1 suggests variable TAAR1 function that may be dependent on the state of macrophage polarization, thereby implicating TAAR1 as a novel target through which inflammatory cellular processes such as migration and proliferation, both of which are associated with the neuroinflammation occurring in an active MS lesion. From a clinical and potentially MS-relevant perspective, TAAR1 is now established as a viable target for human pharmacotherapy of disorders of the CNS [7,8].

An additional observation arising from our results (Figure 2) is the subcellular protein expression pattern of TAAR1 from the nucleus into the cytoplasm upon pro-inflammatory macrophage stimulation. This finding is consistent with previous results whereby TAAR1 localization is primarily observed within the intracellular environment [20,24,25,26,27,28]. Interestingly, there has been no consensus as to the exact location of TAAR1 within the cell. A recent study has shown that following serum-starving, TAAR1 shifts from a perinuclear localization to the cilia of mouse thyroid epithelial cells in order to facilitate TAAR1 interaction with the extracellular environment [27], indicating that TAAR1 sub-cellular localization is dependent on environmental (extracellular) signals. A similar situation could be occurring in pro-inflammatory macrophages, where the function of TAAR1 under these conditions requires trafficking to an interface with the extracellular environment. Other studies have primarily associated TAAR1 localization with intracellular membrane structures such as the golgi apparatus [20,31]. Higher resolution, real-time imaging studies are required to further clarify the consequences of variable sub-localization of TAAR1 and the signals mediating receptor trafficking.

Finally, we provide the first visualization of TAAR1 protein in human brain tissue with immunohistochemical analysis of a post-mortem MS patient brain section (Figure 3). As previously performed in our lab, we first identified mixed active/inactive MS lesions with H&E (Figure 3A), LFB (Figure 3B), and CD68 (Figure 3C) staining via brightfield microscopy [22]. A mixed active/inactive lesion features an “active,” and expanding, border region rich with pro-inflammatory peripheral macrophages and CNS-resident microglia, in addition to an inactive and demyelinated center composed of glial scar tissue [32]. The area surrounding the mixed/inactive lesion is classified as NAWM on the basis of normal appearing myelination and lack of immune cell aggregation [32]. The lesion center region showed little staining for TAAR1 and is consistent with noticeably fewer macrophages/microglia and metabolically inactive scar tissue (Figure 3F). Meanwhile the NAWM had noticeably more TAAR1 staining (Figure 3D), however virtually no co-localization with the macrophage/microglia marker IBA-1 was observed (Figure 3G). Interestingly, the most pronounced colocalization of IBA-1 with TAAR1 was observed within the inflamed border region of the MS lesion (Figure 3E). More specifically, we observed macrophages or microglia with particularly prominent TAAR1 staining (Figure 3H) associated in close proximity with blood vessels within this region. This raises the intriguing possibility that TAAR1 protein is upregulated in macrophages (perhaps in contrast to microglia) during the active phase of extravasation and CNS invasion. This would be consistent with previous reports that TAAR1 plays a role in immune cell chemotaxis [16]. In these cells we observed a diffuse intracellular localization, similar to that observed in pro-inflammatory stimulated peripherally-derived macrophages (Figure 2). Within the defined border region, but more distant from the blood vessel, we observed a combination of macrophages/microglia with and without TAAR1 staining (Figure 3I). This further supports our hypothesis that TAAR1 protein is increased in extravasating peripheral macrophages acting with pro-inflammatory function within the border of an MS lesion and will be an area of focus for future investigations.

In conclusion, this communication demonstrates the potential for a novel link between TAAR1 and MS pathophysiology. We observed a statistically significant decrease in TAAR1 mRNA in MS patient monocytes compared to controls, a pronounced shift in sub-cellular localization following pro-inflammatory stimulation in peripheral macrophages, and TAAR1 colocalization with the macrophage and microglia marker IBA-1 within the inflamed border region of an MS lesion. These findings support further study towards the role of TAAR1 in monocyte and macrophage populations and warrants that pharmacological investigation using selective TAAR1 agonists [7,8] in animal models of MS [33].

## 4. Materials and Methods

### 4.1. Human Peripheral Blood Mononuclear Cells & Monocyte-Derived Macrophages

All studies involving human samples received institutional review board approval at Memorial University of Newfoundland (Health Research Ethics Authority) and strictly followed Canadian Institute of Health Research (CIHR) guidelines. Patient recruitment was performed both within MS and outpatient neurology clinics by a registered nurse and/or a physician. Patient demographics are provided in Table 1, Table 2 and Table 3. Following informed consent, whole peripheral blood was collected from MS patients, NIND patients, and healthy study participants (controls) in EDTA-coated tubes. CD14+ monocytes were collected from an independent MS patient cohort compared to the MS and NIND patient samples used for the whole PBMC analysis.

For PBMC and monocyte isolation, the following solutions were used: PBMC medium (pH = 7.4) consisting of Roswell Park Memorial Institute (RPMI) 1640 medium (ThermoFisher/Life Technologies; Waltham, MA, USA), 10% heat-inactivated fetal bovine serum (HI FBS) (USA Sourced, Corning, NY, USA), 1× GlutaMAX (Thermofisher/Life Technologies; Waltham, MA), and 1× penicillin/streptomycin (P/S) (Thermofisher/Life Technologies; Waltham, MA). Magnetic-activated cell sorting (MACS) buffer (pH = 7.4) consisting of sterile phosphate buffered saline (PBS; 10 mM PO_4_, 137 mM NaCl, pH = 7.4, consistent throughout all studies) 2 mM EDTA and 0.5 % FBS was used for CD14+ cell separation.

Whole blood was pooled from two 10 mL BD Vacutainer samples from each donor into a 50 mL conical tube. The original tubes were then rinsed with 5 mL of PBS, and the contents were added to the corresponding 50 mL conical tube. The conical tube was then filled to 35 mL with additional PBS. SepMate™ tubes (StemCell Technologies; Vancouver, BC) were used to isolate PBMCs. The lower chamber of the SepMate™ tube was filled with 15 mL of Ficol-Hypaque (ThermoFisher/Life Technologies; Waltham, MA) and the 35 mL of blood/PBS mixture was carefully added to the upper chamber. Each SepMate tube was centrifuged at 1200× *g* for 10 min to separate the contents of the tube. PBMCs were carefully collected/scraped from the side of the tube and transferred to a fresh 50 mL conical tube, which was then brought up to 50 mL with PBS. This PBMC enriched solution was then centrifuged at 300× *g* for 15 min and the supernatant was aspirated. From here, the PBMC pellet was either resuspended in PBMC medium and counted prior to cryopreservation or resuspended in 20 mL of MACS buffer and counted prior to monocyte isolation. For counting and viability check, 10 µL of a cell suspension was added to 10 µL of trypan blue (Sigma Aldrich; St. Louis, MO, USA) and live cell density determined with a Countess™ II Automated Cell Counter (Applied Biosystems; Waltham, MA, USA).

For monocyte isolation, the sample was brought to 50 mL with MACS buffer and centrifuged at 300× *g* for 10 min. The supernatant was aspirated and the pellet was resuspended in 80 µL/1 × 10^7^ cells of MACS buffer. Anti-CD14 antibody coated magnetic beads (20 µL; Miltenyi Biotec; Bergisch Gladbach, Germany) were added and the samples were incubated at 4 °C for 15 min and the MACS column washed with 3 mL of MACS buffer. Following incubation, the samples were washed with 20 mL of MACS buffer and centrifuged at 300× *g* for 10 min. The supernatant was aspirated and the PBMCs were resuspended in MACS buffer to 1× 10^7^ cells/mL (1 mL minimum). The prepared cell suspension was then poured directly through the MACS column and flow through was collected in a new 15 mL conical tube. The column was washed three times with 3 mL of MACS buffer and the combined flow throughs discarded. The MACS column was then carefully removed from the magnet and placed into a fresh 15 mL conical tube. MACS buffer (5 mL) was added to the column and quickly plunged to expel the separated CD14+ monocytes from the column. A cell count was conducted as previously described and the sample was centrifuged at 300× *g* for 15 min. CD14+ monocytes were then resuspended to 5 × 10^5^ cells/mL in macrophage medium consisting of RPMI, 10% HI-FBS, 1 × P/S, 1 × GlutaMAX, and macrophage colony-stimulating factor (M-CSF; 25 ng/mL) and plated in the appropriate experimental vessel. Following incubation at 37 °C for 3 days, a half medium change was performed. The cells were then used for experimentation within 3–4 days.

### 4.2. RNA Isolation, Reverse Transcription, and Quantitative Polymerase Chain Reaction

Human PBMCs from MS patients, NIND patients, or healthy volunteers as well as CD14+ monocytes from MS patients or healthy volunteers were lysed with QIAzol^®^ reagent (Qiagen; Hilden, Germany) and used for RNA extraction with the RNeasy^®^ Micro Kit (Qiagen; Hilden, Germany). RNA extraction was carried out according to the protocol provided by the manufacturer. RNA concentration was determined using a Nanodrop 1000 Spectrophotometer (Fisher Scientific; Waltham, MA, USA). The High Capacity cDNA Reverse Transcription Kit (Applied Biosystems; Waltham, MA, USA) was used to generate cDNA according to the protocol provided by the manufacturer. TaqMan^®^ Fast Universal PCR Master Mix (Applied Biosystems; Waltham, MA) was used to conduct the polymerase chain reaction (PCR). The human TaqMan^®^ FAM-TAAR1 (Hs00373229_s1) and VIC-hGAPDH (Hs02786624_g1) probes and primers (Applied Biosystems; Waltham, MA) were used to assess TAAR1 expression in human cells of interest. RT-qPCR was performed with the Applied Biosystems^®^ ViiA 7 Real-Time PCR System with the following parameters: 2 min hold at 50 °C followed by a 2 min hold at 95 °C before proceeding to 40 cycles of 95 °C (3 s) and 60 °C (30 s). No-template and reverse transcriptase negative controls were included. Analysis was conducted on QuantStudio™ Software by Applied Biosystems and fold changes were determined using the ΔΔCT method [34].

### 4.3. Immunocytochemistry

Human CD14+ monocytes were seeded at 100,000 cells/well in 8-well Permanox^®^ Chamber slides (Lab-Tek^®^; Fisher Scientific; Waltham, MA) and were grown under standard culturing conditions (Section 4.1). For pro-inflammatory stimulation, the culture medium was changed into a serum-free environment (RPMI, 1× GlutaMAX, and 1× P/S) and then treated with lipopolysaccharide (LPS) (Sigma Aldrich, L2880; St. Louis, MO, USA) at 100 ng/mL for 24 h. Following stimulation, macrophages were fixed with 2% formalin for 15 min at room temperature. Fixed macrophages were then washed with 500µL of PBS and permeabilized with 500 µL of 0.3% triton X-100 (ThermoFisher; Waltham, MA, USA) in PBS for 10 min at room temperature. Following permeabilization, cells were blocked with 500 µL of blocking buffer (pH = 7.4; 10% goat serum (ThermoFisher; Waltham, MA) in PBS with 0.1% bovine serum albumin (ThermoFisher; Waltham, MA), 0.3% triton X-100 (ThermoFisher; Waltham, MA), and 0.05% TWEEN^®^ (ThermoFisher; Waltham, MA) for 1 h at room temperature. Next, a previously validated [20] monoclonal mouse anti-human TAAR1 antibody (Hoffman-La Roche, Switzerland) was used at a working concentration of 16.2 µg/mL in immunofluorescence (IF) buffer (pH = 7.4; PBS with 0.1% bovine serum albumin (ThermoFisher; Waltham, MA), 0.3% triton X-100 (ThermoFisher; Waltham, MA), and 0.05% TWEEN^®^ (ThermoFisher)) and incubated with the cells for 16–18 h at 4 °C in the dark. IF buffer without the antibody was used as a “no-primary” negative control. Following primary antibody incubation, the macrophages were washed 3 times for 15 min with 500 µL of PBS. Following washing, macrophages were incubated with 500µL of the secondary antibody solution (PBS with a goat anti-mouse IgG AlexaFluor™ 594-congugated antibody (1:500, Life Technologies; Waltham, MA, A11005), AlexaFluor™ conjugated phalloidin (2 drops/mL, Invitrogen™). and 4′,6-diamidino-2-phenylindole (DAPI; 1:1000)) for 1 h at room temperature in the dark. Following secondary antibody incubation, macrophages were washed 3 times for 5 min with 500 µL of PBS. Next, the chambers were carefully removed, and the slide was coated with 2 drops of Fluoromount—G before applying a coverslip. The slide was then sealed with nail polish and stored in the dark at 4 °C until imaging with a Zeiss AX10 fluorescent microscope at 40× and 63× magnification.

### 4.4. Histology and Immunohistochemistry

Formalin-fixed paraffin-embedded human brain sections from mixed active/inactive MS lesions were used for in situ studies following a next-of-kin consented autopsy and was approved by the Newfoundland Health Research Ethics Board. Sections were subjected to heat-induced antigen retrieval using sodium citrate buffer (10 mM, pH = 6.0) and blocked with PBS containing 10% horse serum (ThermoFisher; Waltham, MA) for 1 h. For primary antibody incubation, sections were incubated overnight at 4–8 °C with an antibody solution containing the TAAR1 antibody (16.2 µg/mL) and a chicken anti-human IBA-1 antibody (20 µg/mL, Aves Labs, AIF101; Davis, CA, USA) in PBS with 10% horse serum. Following overnight incubation, sections were washed five times with PBS for 5 min. For secondary antibody incubation, sections were incubated at 4–8 °C for 1 h with a secondary antibody solution containing a goat anti-mouse IgG AlexaFluor™ 594 conjugated antibody (1:500, Invitrogen, A11005; Waltham, MA, USA), a goat anti-chicken IgG AlexaFluor™ 488 conjugated antibody (1:500, Invitrogen, A32931; Waltham, MA), and DAPI (1:1000) diluted in PBS. Following the secondary antibody incubation, sections were washed five times for 5 min with PBS and coated with 2 drops of Fluoromount—G before applying a coverslip. The sections were stored in the dark at 4 °C prior to imaging with a Zeiss AX10 fluorescent microscope at 10× and 40× magnification.

### 4.5. Statistical Analysis

Statistical analyses were performed using GraphPad Version 9.1.1. Data is presented in the form of mean ± standard error of the mean (SEM). The Kruskal–Wallis one-way analyses of variance (ANOVA) test with Dunn’s post hoc multiple comparisons test was used where applicable, following determination of variance by the Brown-Forsythe ANOVA test. Unpaired t test was used when recommended for pairwise comparison. *p* < 0.05 was considered significant.

## Figures and Tables

**Table 1:** Study participant demographic data (*TAAR1* expression in whole PBMC cohort).

	Number of Participants	Age (Years)	Female:Male Ratio
NIND	10	47 ± 1	2.67
MS	14	44 ± 9	1.85
Controls	15	51 ± 7	1.16

**Table 2:** Participant noninflammatory neurological disorders (NIND).

NIND	Number of Participants (10)
Idiopathic intracranial hypertension	5
Upper motor neuron disease	1
Visual migraine phenomenon	1
Spasticity	1
Ataxia	1
Neurogenic bladder	1

**Table 3:** Study participant demographic data (TAAR1 expression in CD14+ monocytes cohort).

	Number of Participants	Age (Years)	Female:Male Ratio
MS	11	42 ± 11	1.75
Controls	9	38 ± 11	2

## Supplementary Materials

The following are available online at https://www.mdpi.com/article/10.3390/ijms222111576/s1.
