# Supplementary material for: TAAR1 Expression in Human Macrophages and Brain Tissue: A Potential Novel Facet of MS Neuroinflammation
Source: Int J Mol Sci. 2021 Oct 27;22(21):11576. doi: 10.3390/ijms222111576 (PMC8584001; doi:10.3390/ijms222111576)
Supplement: Supplementary file 1 [file ijms-22-11576-s001.zip › ijms-1420244-supplementary.pdf]

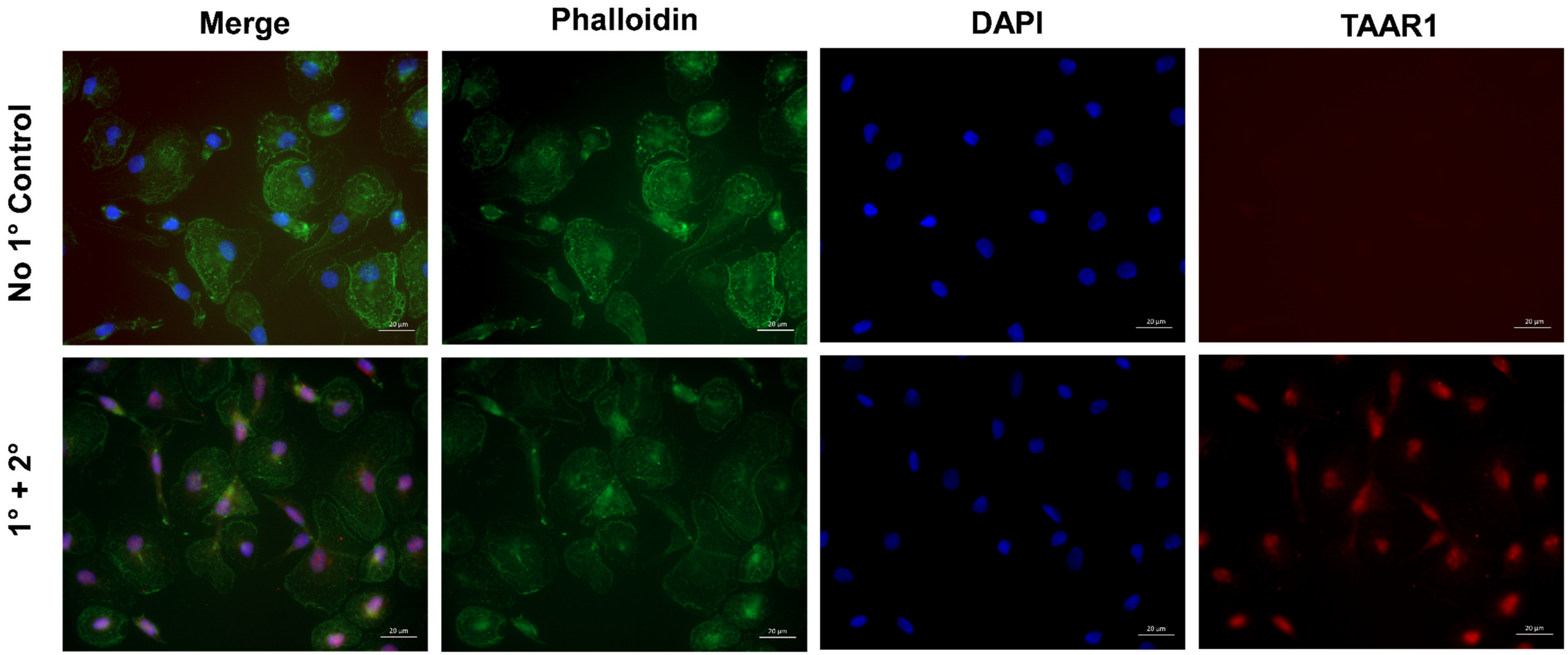

Supplementary Figure S1. anti-TAAR1 antibody selectivity validation in basal CD14+ monocyte-derived macrophages from healthy volunteers.

TAAR1 protein was visualized with a validated anti-human TAAR1 mouse primary antibody combined with a goat anti-mouse IgG AlexaFluor™ 594-conjugated secondary antibody (red). Nuclei were visualized via DAPI staining (blue) and actin with AlexaFluor™ 488-conjugated phalloidin (green). The top row contains macrophages stained with an antibody solution lacking the primary anti-TAAR1 antibody. All images were taken with the Zeiss AX10 fluorescent microscope at 63X magnification generated in the built-in Zeiss software.
